# Cerebral perfusion alterations in patients with trigeminal neuralgia as measured by pseudo-continuous arterial spin labeling

**DOI:** 10.3389/fnins.2022.1065411

**Published:** 2022-12-16

**Authors:** Qianling Zhou, Meng Li, Qisen Fan, Feng Chen, Guihua Jiang, Tianyue Wang, Qinmeng He, Shishun Fu, Yi Yin, Jinzhi Lin, Jianhao Yan

**Affiliations:** ^1^The Second School of Clinical Medicine, Southern Medical University, Guangzhou, China; ^2^Department of Medical Imaging, Guangdong Second Provincial General Hospital, Guangzhou, China; ^3^Department of Anesthesiology, The First Affiliated Hospital of Guangzhou Medical University, Guangzhou, China; ^4^Department of Neurosurgery, Guangdong Second Provincial General Hospital, Guangzhou, China

**Keywords:** trigeminal neuralgia, chronic pain, arterial spin labeling, cerebral blood flow, functional MRI, brain

## Abstract

**Background:**

Accumulating evidence suggests that trigeminal neuralgia (TN) causes structural and functional alterations in the brain. However, only a few studies have focused on cerebral blood flow (CBF) changes in patients with TN. This study aimed to explore whether altered cerebral perfusion patterns exist in patients with TN and investigate the relationship between abnormal regional CBF (rCBF) and clinical characteristics of TN.

**Materials and methods:**

This study included 28 patients with TN and 30 age- and sex-matched healthy controls (HCs) who underwent perfusion functional MRI (fMRI) of the brain using pseudo-continuous arterial spin labeling (pCASL) in the resting state. The regions of significantly altered CBF in patients with TN were detected using group comparison analyses. Then, the relationships between the clinical characteristics and abnormal rCBF were further investigated.

**Results:**

Compared to the control group, patients with TN exhibited increased rCBF, primarily in the thalamus, middle frontal gyrus (MFG), and left insula. Furthermore, the CBF values of the thalamus were negatively correlated with the pain intensity of TN and positively correlated with pain duration in patients with TN.

**Conclusion:**

Primary alterations in rCBF in patients with TN occurred in different brain regions related to pain, which are involved in cognitive-affective interaction, pain perception, and pain modulation. These results indicate that non-invasive resting cerebral perfusion imaging may contribute complementary information to further understanding the neuropathological mechanism underlying TN.

## 1 Introduction

Trigeminal neuralgia (TN) is a clinically common chronic neuropathic pain disorder characterized by recurrent paroxysmal electric shock-like pain in the trigeminal distribution (Zhang et al., 2018). TN is one of the most painful and stubborn illnesses. Over time, pain may become more frequent and sustained, resulting in anxiety and depression, and ultimately lowering quality of life (Zakrzewska et al., 2017; Zhang et al., 2018). It is estimated that TN has an incidence of 2–5 per 100,000 persons, with an even higher prevalence among aging demographics (Mueller et al., 2011; De Toledo et al., 2016). Unfortunately, approximately 20–30% of patients with TN report unsatisfactory treatment outcomes (Zakrzewska and Akram, 2011; Cruccu et al., 2016; Lv et al., 2022), highlighting a clear and urgent need to understand the pathogenesis of TN better and develop more effective treatment strategies.

Several studies have found underlying brain anomalies associated with chronic pain, including alterations in brain function, structure, chemistry, and cerebral perfusion (Simons et al., 2014; Kregel et al., 2015; Kumbhare et al., 2017; Jung et al., 2019; Lee et al., 2019). Recent studies have reported that patients with TN have structural and functional brain alterations (Obermann et al., 2013; Henssen et al., 2019; Yan et al., 2019; Zhang et al., 2021). In addition, researchers have revealed several critical brain regions of TN, including the thalamus, insular cortex, cingulate cortex, and dorsolateral prefrontal cortex (DLPFC), which led to the hypothesis that pain-related central regulation may be at work in TN. Cerebral perfusion is closely associated with cerebral metabolism. One method to assess cerebral blood flow (CBF) is positron emission tomography, which uses radioactive tracers to map cerebral glucose metabolism and detect significant changes in glucose metabolic patterns secondary to chronic pain (Kulkarni et al., 2007; Tu et al., 2009). Alternatively, magnetic resonance imaging (MRI)-based mapping of CBF using arterial magnetic spin labeling (ASL) has shown potential in non-invasively studying pain states including fibromyalgia, post-herpetic neuralgia, persistent low back pain, post-surgical pain, and osteoarthritis (Howard et al., 2011, 2012; Wasan et al., 2011; Liu et al., 2013; Shokouhi et al., 2016). ASL is a technique for evaluating resting-state CBF that could be considered a marker for clinical assessment of functional activation. It provides a direct measure of regional CBF (rCBF) without requiring complex computations (Detre et al., 1992). ASL perfusion functional MRI (fMRI) has been used to detect CBF for a variety of neurological and psychiatric diseases owing to its better resolution and accurate localization (Ho, 2018; Zeng et al., 2021). However, only a few CBF studies have investigated chronic pain, with even less research conducted on TN. Therefore, ASL MRI may provide additional information in understanding the pathogenesis of TN.

As a non-invasive technique for measuring CBF, ASL perfusion MRI can be classified into three types: pulsed ASL, continuous ASL, and pseudo-continuous ASL (pCASL). Compared to continuous ASL, pCASL uses a series of very short radiofrequency (RF), and gradient pulses that provide much lower RF energy deposition and does not require special hardware. Moreover, pCASL has a better signal-to-noise ratio (SNR) in comparison to pulsed ASL. Therefore, the pCASL approach provides a good balance between labeling efficiency and SNR (Wu et al., 2007).

This study aimed to use pCASL to identify differences in rCBF between patients with TN and matched healthy controls (HCs). Additionally, we explored the relationship between the clinical features of TN and abnormal rCBF. We hypothesized that patients with TN may have aberrant rCBF in some brain regions involved in specific cortical functions including perception, sensorimotor, and emotional processing. rCBF in the abnormal brain regions may be associated with the clinical characteristics of patients with TN.

## 2 Materials and methods

### 2.1 Subject population and recruitment

Patients with TN were recruited from the Department of Neurosurgery at Guangdong Second Provincial General Hospital from January 2021 to July 2022. All patients with TN were diagnosed according to the International Classification of Headache Disorders criteria (version III) (Marcel, 2018). The inclusion criteria for patients were as follows: (a) age range of 18–70 years old; (b) right-handedness; (c) no brain trauma or neurological disease; (d) no history of alcoholism, intake of psychiatric medications, or substance abuse; and (e) no MRI contraindications. Patients with TN were excluded as follows: (a) patients with chronic pain or neural-associated disorders other than TN; (b) patients with a history of craniocerebral surgery; or and (c) patients with contraindications to MRI (e.g., cardiac pacemaker, claustrophobia, or pregnancy). Overall, 28 eligible patients with TN were included in this study according to the above criteria. We recorded each patient with TN medication status and disease duration and evaluated the extent of their neuralgia according to the visual analog scale (VAS). A psychiatrist quantified anxiety- and depression-related symptoms of each patient based on the Hamilton Anxiety Rating Scale (HAMA) and the 24 items Hamilton Depression Rating Scale (HAMD).

For comparison, 30 age- and sex-matched HCs were recruited to participate in the study based on the following criteria: (a) age range of 18–70 years old; (b) right-handedness; (c) no neurological disorders; (d) absence of alcoholism, intake of psychiatric medications or substance abuse; (e) no history of acute or chronic painful conditions during the screening visit; and (f) no MRI contraindications.

### 2.2 Image acquisition

The imaging data were acquired on a 3.0-T Ingenia MR scanner using a 32-channels head coil (Philips Healthcare, Best, Netherlands). First, high-resolution three-dimensional brain structural images were obtained using a fast field echo pulse sequence. The parameters of this scan were as follows: repetition time (TR)/echo time (TE) = 7.9/3.6 ms; field of view (FOV) = 256 × 256; matrix = 256 × 256; flip angle (FA) = 9^°^; slice thickness = 1.0 mm with no gap; 185 transverse slices. Anatomical images were used to register functional images and modify the anatomical atlas. Furthermore, 3D fast spin-echo collection with pCASL sequences was used to perform resting-state perfusion imaging (TE/TR = 4,155/33 ms, FOV, 240 mm × 240 mm × 240 mm; matrix, 64 × 59; flip angle, 90^°^; post-labeling duration, 2,000 ms; the number of slices, 20; 6 mm thickness).

Simultaneously, a conventional MRI was obtained to rule out any anatomical abnormalities. Two qualified radiologists diagnosed each participant to ensure that there were no structural abnormalities in their brains.

### 2.3 Data pre-processing

Arterial magnetic spin labeling images were processed on a Philips post-processing workstation, and CBF was quantified based on the formula below:


$$\text{CBF}=\frac{6,000\cdot \lambda \,\left({\text{SI}}_{\text{control}}-{\text{SI}}_{\text{label}}\right)\cdot e^{\frac{\text{PLD}}{T_{1,\text{blood}}}}}{2\cdot \alpha \cdot T_{1,\text{blood}}\cdot S\,I_{P\,D}\cdot \left(1-e^{-\frac{\text{LT}}{T_{1,\text{blood}}}}\right)}\,\left[m\,l/100\,g/\text{min}\right]$$

This calculation was based on the assumption of 1,650 ms for *T*1 of blood (*T*_1, blood_) at 3.0 T, a partition coefficient (λ) of 0.9, a labeling efficiency (α) of 0.85, a post-labeled delay (PLD) of 2,000 ms, and a labeling duration (LT) of 1,800 ms. SI_control_ and SI_label_ represent the time-averaged signal intensity of control label images and the time-averaged signal intensity of the label images, respectively. SI_*PD*_ represented the signal intensity in a proton density-weighted image.

We used SPM12 software^1^ to normalize the CBF maps to the Montreal Neurological Institute (MNI) standard space. First, to create individual T1’ brain maps, the individual CBF brain maps were co-registered with their own 3D T1-weighted structural image. Second, the T1’ brain maps were segmented into gray matter, white matter, and cerebrospinal fluid. In this step, the transformation parameters were obtained. Third, each CBF map was normalized to MNI space using a voxel size of 2 mm with reference to the transformation parameters estimated from step two. Then, to reduce the influence of CBF values by scanning parameters and individual hemodynamic variability, we used the DPABI software^2^ package based on MATLAB R2020a to standardize the data from CBF maps obtained after spatial normalization. Two different normalization methods were used to calculate the CBF maps in this study. In the first method, the *z*-transformation of CBF values for each voxel in each individual was performed using the following equation (He et al., 2019; Chen et al., 2022):


$$z\,{\text{CBF}}_{\text{vox}}=\frac{{\text{CBF}}_{\text{vox}}-{\text{CBF}}_{\text{gmean}}}{{\text{CBF}}_{\text{gstd}}}$$

CBF_vox_ is single voxel CBF, CBF_gmean_ represents the mean CBF of the whole brain within the gray matter mask, and CBF_gstd_ is the standard deviation of the whole brain CBF. In the second method, mean division normalization was performed such that the CBF of each voxel was divided by the average CBF in the whole brain (Xu et al., 2021). Finally, the resulted CBF maps were smoothed with a full-width half-maximum Gaussian kernel of 6 mm.

### 2.4 Statistical analysis

The age and education level information in this study conformed to normal distribution. The independent sample *t*-test was used to analyze the differences in age and education, while the Chi-square test was performed to estimate differences in sex using SPSS 25 (IBM, Armonk, NY, United States). Significant differences were determined at *P* < 0.05.

Using two-sample *t*-tests in the DPABI toolkit with sex, age, and years of education as covariates, we compared the voxel-level CBF maps between the TN and HC groups. Multiple comparison correction was performed using Gaussian random field (GRF) theory, with a voxel value of *P* < 0.001 and a corrected cluster significance of *P* < 0.05. In addition, we used a more liberal threshold to detect differences between the two groups, which used the cluster level *P* < 0.05 with an uncorrected cluster forming threshold of *P* < 0.001.

For significantly abnormal brain regions, we extracted the average CBF values of each cluster separately and then conducted Pearson correlation analysis to investigate the relationship between CBF values and disease duration. Moreover, we used Spearman correlation analysis to evaluate the correlation between the CBF values and pain intensity, HAMD score, and HAMA score in patients with TN.

## 3 Results

### 3.1 Demographics and clinical data

Table 1 shows the demographic and clinical characteristics of all patients with TN and those of HCs. No significant differences were found between the TN and HC groups concerning sex (*P* = 0.849), age (*P* = 0.053), and educational attainment (*P* = 0.173). The TN group experienced pain for an average of 6.51 years.

**TABLE 1:** Demographics and clinical data of patients with trigeminal neuralgia (TN) and healthy controls (HCs).

Characteristics	TN (*n* = 28)	HC (*n* = 30)	*P*-value
Age (years)	52.9 ± 11.8	47.5 ± 8.9	0.053^a^
Sex (female/male)	18/10	20/10	0.849^b^
Education (years)	10.4 ± 3.7	11.6 ± 3.1	0.173^a^
Affected side	7 left/21 right	–	–
Duration of pain (years)	6.5 ± 5.0	–	–
VAS score	8.2 ± 1.7	–	–
HAMD score	13.0 ± 6.8	–	–
HAMA score	12.9 ± 6.7	–	–
Medication (CBZ/GBP/PGB)	25/2/1	–	–

TN, trigeminal neuralgia; HC, healthy control; VAS, visual analog scale; HAMD, Hamilton Depression Rating Scale; HAMA, Hamilton Anxiety Rating Scale; CBZ, carbamazepine; GBP, gabapentin; PGB, pregabalin.

^a^Independent sample *t*-test.

^b^Chi-square test.

### 3.2 Comparison of cerebral blood flow between groups

**FIGURE 1:**
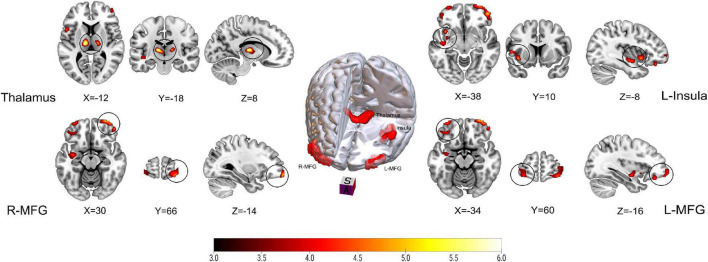
Results on altered regional cerebral blood flow (rCBF) in patients with trigeminal neuralgia (TN) compared with healthy controls (HCs) using *z*-transformation method. The color bar represents *Z*-values. Red clusters indicate significantly increased cerebral blood flow (CBF) in patients with TN. The statistical significance of group differences was determined using Gaussian random field (GRF) correction (voxel *P*-value < 0.001, cluster *P*-value < 0.05). MFG, Middle frontal gyrus; L (R), left (right) hemisphere.

Figure 1 and Table 2 show the rCBF differences between patients with TN and matched HCs using the *z*-transformation method. Patients with TN exhibited increased rCBF, primarily in the thalamus (*P* < 0.001), left insula (*P* = 0.003), right middle frontal gyrus (MFG) (*P* = 0.002), and left MFG (*P* = 0.012) (Table 3). However, regions with decreased CBF in the TN group were not detected using GRF correction with a voxel value of *P* < 0.001 and a corrected cluster significance of *P* < 0.05.

**TABLE 2:** Brain regions that showed significant increases in cerebral blood flow (CBF) in patients with trigeminal neuralgia (TN) compared to matched healthy controls (HCs).

Anatomical regions	Cluster size (ml)	Peak MNI coordinate			*Z* score
		*X*	*Y*	*Z*	
**Z-transformation**					
Thalamus	3.4	–12	–18	8	6.0
Insula, L	3.6	–38	10	–8	4.9
Middle frontal gyrus, R	6.8	30	66	–14	5.3
Middle frontal gyrus, L	3.6	–34	60	–16	4.5
**Mean division**					
Thalamus	3.7	–12	–18	8	6.2
Insula, L	2.8	–50	12	6	5.0
Middle frontal gyrus, R	6.5	30	66	–14	5.2

All regions survived Gaussian random field correction with a voxel value of *P* < 0.001 and a corrected cluster significance of *P* < 0.05. MNI, Montreal Neurological Institute; L, left hemisphere; R, right hemisphere.

**TABLE 3:** Regional cerebral blood flow (rCBF) values of all participants.

Anatomical regions	TN group (*N* = 28)	HC group (*N* = 30)	*P*
**Z-transformation**			
Thalamus	0.146 (0.264)	–0.198 (0.234)	<0.001
Left insula	0.599 (0.261)	–0.126 (0.193)	0.003
Right middle frontal gyrus	–0.004 (0.296)	–0.270 (0.331)	0.002
Left middle frontal gyrus	–0.513 (0.313)	–0.279 (0.384)	0.012
**Mean division**			
Thalamus	1.079 (0.134)	0.903 (0.113)	<0.001
Left insula	1.145 (0.164)	1.049 (0.125)	0.015
Right middle frontal gyrus	0.774 (0.155)	0.629 (0.179)	0.002

All regions survived gaussian random field correction with a voxel value of *P* < 0.001 and a corrected cluster significance of *P* < 0.05. All regions are provided as mean (standard deviation). TN, trigeminal neuralgia; HC, healthy control.

**FIGURE 2:**
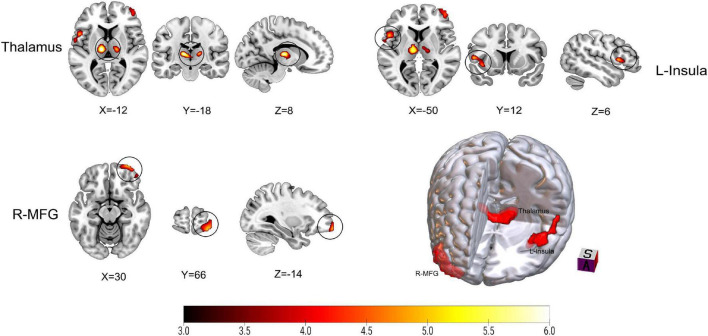
Results on altered regional cerebral blood flow (rCBF) in patients with trigeminal neuralgia (TN) compared with healthy controls (HCs) using mean division method. The color bar represents *Z*-values. Red clusters indicate significantly increased cerebral blood flow (CBF) in patients with TN. The statistical significance of group differences was determined using Gaussian random field (GRF) correction (voxel *P*-value < 0.001, cluster *P*-value < 0.05). MFG, Middle frontal gyrus; L (R), left (right) hemisphere.

**FIGURE 3:**
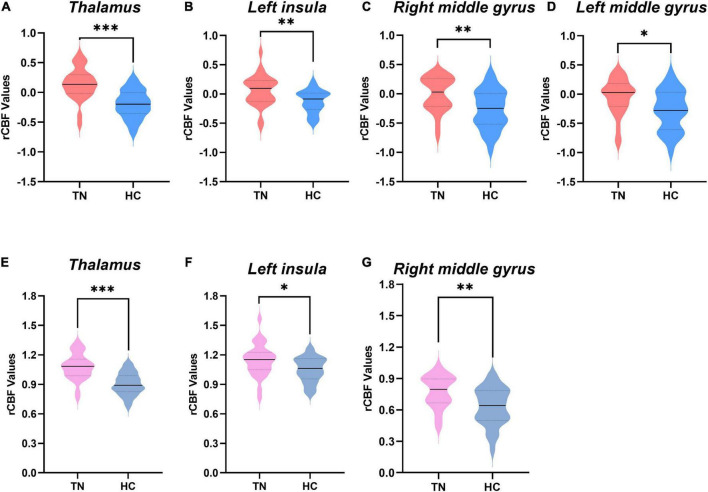
Comparison of regional cerebral blood flow (rCBF) values between the two groups. **(A–D)** The rCBF values were obtained using *z*-transformation. **(E–G)** The rCBF values were calculated by the mean division method. rCBF values of patients with trigeminal neuralgia (TN) (red or pink) and healthy controls (HCs) (blue or gray) in the thalamus, middle frontal gyrus (MFG), and left insula are displayed using violin plots. *P*-values less than 0.001 are indicated by three asterisks. Double asterisks represent *P*-values greater than 0.001 and less than or equal to 0.01. one asterisk represents *P*-values less than 0.05 but greater than 0.01. Solid horizontal lines indicate group medians. TN, trigeminal neuralgia; HC, healthy control.

Figure 2 and Table 2 show the differences in rCBF between patients with TN and matched HCs using the mean division method. Compared with the results obtained by the *z*-transform method, patients with TN exhibited similar activation clusters except for the left MFG, including increased rCBF in the thalamus (*P* < 0.001), left insula (*P* = 0.015), and right MFG (*P* = 0.002) (Table 3). It can be seen that the cluster sizes of activated thalamus and right MFG and their intergroup significance were similar in both approaches, while the left insula was greater significance and larger cluster sizes in the *z*-transformation method. Figure 3 presents the rCBF values for the TN and HC groups.

In addition, we detected some additional cerebral regions with decreased rCBF in patients with TN, including the right post-central gyrus, right pre-central gyrus, right middle temporal gyrus, right superior temporal gyrus, and left calcarine, which were taken at cluster level *P* < 0.05 with an uncorrected cluster forming threshold of *P* < 0.001 (Supplementary Figure 1 and Table 1).

### 3.3 Correlations between cerebral blood flow and clinical data

**FIGURE 4:**
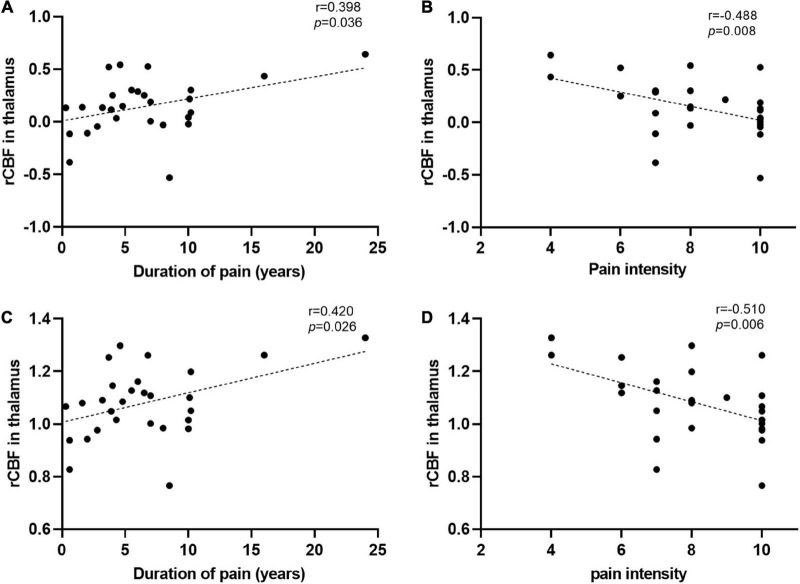
Significant correlations between the regional cerebral blood flow (rCBF) alterations and clinical data. **(A,B)** The rCBF values were obtained using *z*-transformation. **(C,D)** The rCBF values were calculated by the mean division method. **(A,C)** Pearson correlation analysis showed that the increase of rCBF in the thalamus of patients with trigeminal neuralgia (TN) was correlated with their disease duration. **(B,D)** Spearman correlation analysis revealed that the increased thalamic blood flow in patients with TN was associated with pain intensity. L (R), left (right) hemisphere.

Correlation analysis showed that both CBF normalized by *z*-transformation and CBF normalized by the mean division demonstrated a positive correlation between thalamic CBF and disease duration and a negative correlation between pain intensity in patients with TN (Figure 4). In the correlation analysis, the strength of clinical correlation and *P*-value of thalamic CBF values normalized by using the mean division method were slightly more significant than the those normalized using the *z*-transformation method. However, no negative or positive correlations were observed between the CBF values of the other clusters listed in Table 2 and the duration or intensity of pain. Furthermore, other clinical measures, including years of education, HAMD score, and HAMA score, did not significantly correlate with CBF values in the abnormal region.

## 4 Discussion

In this study, we used pCASL for the first time to explore alterations in blood flow in the brains of patients with TN. rCBF maps were calculated based on two different normalization methods. By comparing the results of the two methods, we found that although the *z*-transformation method might have detected brain regions with abnormal rCBF more sensitively than the mean division method, the latter detected abnormal brain regions with slightly stronger clinical correlation than the former. Both of these methods consistently detected significant differences in rCBF in patients with TN compared with HCs, which were mainly located in the insula, thalamus, and MFG. These results are consistent with those of previous studies based on structural and fMRI (Henssen et al., 2019). In addition, changes in CBF values in the thalamus were significantly associated with the duration and intensity of pain, further suggesting their clinical relevance.

The thalamus, which shows increased rCBF in patients with TN, is a crucial relay station for the transmission of noxious information to the cerebral cortex (Groh et al., 2018). Several previous studies have reported increased activity in the thalamus of patients with TN (Moissetl et al., 2011; Wang et al., 2015; Yan et al., 2019). In particular, one of our previous blood oxygen level-dependent fMRI studies on TN observed increased dynamic regional homogeneity in the thalamus of patients with TN (Yan et al., 2019). Moreover, several studies have associated chronic pain with increased thalamic blood flow (Howard et al., 2011, 2012). For instance, Liu et al. (2013) found that rCBF in the thalamus was significantly increased and highly correlated with pain intensity in post-herpetic neuralgia patients using ASL. In addition, Ushida et al. (2010) found increased CBF within the thalamus in patients with complex regional pain syndrome in the early stage (i.e., less than 12 months) but reduced CBF as the disease progressed or after therapy. A previous study showed that rCBF alterations in the thalamus were mainly due to pain-induced changes in endogenous opioid neurotransmission (Wey et al., 2014). Therefore, we speculate that elevated rCBF may signify persistent injury-induced input caused by pain, which is closely associated with the symptoms of TN. Notably, in contrast to the results of several previously mentioned studies, we have observed a negative relationship between rCBF within the thalamus and pain intensity. This discrepancy may be caused by differences in the type, intensity, and duration of spontaneous pain. In addition, we discovered that the rCBF value of the thalamus was positively correlated with the disease duration, which is in accordance with previous findings (Yan et al., 2019). It is possible that the greater the pain intensity in patients with TN, the lower the CBF in the thalamus. The higher CBF in the thalamus may indicate that the pain of patients with TN is less pronounced, thus suggesting the longer duration of the disease. Together, these findings suggest that the thalamus is involved in the development and/or maintenance of chronic pain. Additionally, aberrant thalamic perfusion may be an important characteristic of TN.

In addition, we found that abnormalities also occurred in the left insula in patients with TN. The insula is involved in the affective and sensory dimensions of pain experiences (Lu et al., 2016). A previous review emphasized that the insula may play a role in anxiety regulation (Paulus and Stein, 2006), and Simmons et al. (2011) observed increased activity in the insula of anxiety-prone subjects. In a recent epidemiological study, patients with TN often experienced a terrible fear of triggering pain, which made them vulnerable to negative thoughts such as anxiety and depression (Zakrzewska et al., 2017). In addition to emotional reactions to pain, the insula is a part of the cortical network dedicated to the sensory aspects of pain. Peltz et al. (2011) found that the extent of activation of the insula likely reflects the intensity of a noxious stimulus. Liu et al. (2013) observed that rCBF in the left insula of post-herpetic neuralgia increased, and the degree of CBF in this region correlated with pain intensity using ASL techniques. Moreover, a recent study observed that patients with TN had a significantly thinner insula compared with controls and reversal of gray matter thickness to normal in the insula after effective therapy, further supporting the idea that the insula is potentially involved in the sensory integration and modulation of pain. Therefore, in our study, we speculate that the increased rCBF in the left insula might be associated with the processing of negative emotions, pain perception, and pain modulation in patients with TN.

Interestingly, we also observed that patients with TN exhibited an obvious increase in rCBF in the MFG. The MFG is a crucial part of the DLPFC, which is considered a critical node of networks participating in nociceptive processing and pain modulation. The DLPFC is involved in pain detection, pain suppression, and cognitive and emotional control (Seminowicz and Moayedi, 2017). Freund et al. (2009) found that participants given a task to suppress pain showed increased activation of the bilateral DLPFC during tonic painful stimulation. Bilateral DLPFC activation is linked to reduced unpleasantness of thermal pain (Lorenz et al., 2003). Previous studies on placebo analgesia have also shown that the DLPFC is involved in the suppression of pain, and suppressing DLPFC activity could block placebo analgesia (Krummenacher et al., 2010). A recent study demonstrated that an anticipatory mechanism that modifies frontal attentional processes might cause observationally induced placebo hypoalgesia (Raghuraman et al., 2019). According to these studies, the DLPFC serves as an interface between cognitive processing and pain modulation. Thus, we speculate that activation of the DLPFC in patients with TN may be associated with the control of nociception and possible regulation of pain through anticipatory mechanisms.

Furthermore, using cluster level *P* < 0.05 with an uncorrected cluster forming threshold of *P* < 0.001, we additionally observed decreased rCBF in the right temporal lobe, right post-central gyrus, right pre-central gyrus, and left calcarine. A meta-analysis of structural brain abnormalities in chronic pain has pointed out that the superior temporal gyrus is involved in pain processing (Smallwood et al., 2013). One of our previous studies using dynamic regional homogeneity has shown that TN is associated with abnormal brain function in the pre-central gyrus and middle temporal gyrus (Yan et al., 2019). Moreover, a regional homogeneity study in patients with TN after percutaneous RF thermo coagulation has shown alternations in the right middle temporal gyrus, right calcarine, and left post-central gyrus, which may be related to sensory, affective, and emotional processes (Dou et al., 2016). The observed patterns of decreased rCBF brain regions using a more liberal threshold of *P* < 0.001 (uncorrected) support functional and structural changes in chronic pain from previous studies. Nevertheless, whether these brain regions contribute to the pathogenesis of TN is unclear. Further studies with larger sample sizes should be conducted in the future.

The present study has several limitations. First, our study group was small, which could have affected the accuracy of the results. In the future, we will recruit more patients with TN to verify the findings of our current study. Second, this study used a cross-sectional design, which did not allow us to trace the dynamic development of cerebral perfusion abnormalities in TN. Longitudinal investigations are required in the future to determine the dynamic patterns of these anomalies. Third, all patients with TN in this investigation consumed painkillers, and we cannot rule out the possibility of confounding effects of drugs on CBF analysis.

To the best of our knowledge, this study is the first to use pCASL to detect alterations in rCBF in patients with TN, which were observed mainly in the thalamus, insula, and MFG. These brain regions are primarily involved in cognitive-affective interaction, pain perception, and modulation. The observed patterns of stimulus-related alterations in the brains of patients with TN and their correlation with clinical variables imply that aberrant regional cerebral perfusion may be involved in the patho-physiological mechanisms of TN. This discovery may have a significant contribution to furthering our current understanding of the neuropathological mechanism underlying TN and providing a potential strategy for using imaging markers to objectively evaluate painful conditions in the clinic.

## Data availability statement

The raw data supporting the conclusions of this article will be made available by the authors, without undue reservation.

## Ethics statement

The studies involving human participants were reviewed and approved by the Medical Ethics Committee of Guangdong Second Provincial General Hospital. The patients/participants provided their written informed consent to participate in this study.

## Author contributions

JY and QZ designed the experiment. SF, QH, YY, and TW collected and sorted out the brain imaging data. FC, GJ, and QF helped with imaging data management and processing. JL assisted with acquiring clinical details for the patients. QZ and ML analyzed the data. QZ wrote the manuscript. JY examined and revised the manuscript. All authors reviewed and agreed on the final version.

## Footnotes

1
http://www.fil.ion.ucl.ac.uk/spm

2
http://rfmri.org/DPABI

## Supplementary material

Supplementary Figure 1 Results of altered regional cerebral blood flow (rCBF) in patients with trigeminal neuralgia (TN) compared with healthy controls (HCs). Results were taken at cluster level *P* < 0.05, with a cluster forming threshold of *P* < 0.001 uncorrected. The cold and warm colors indicated that the cerebral blood flow (CBF) value decreased and increased, respectively, in the brain regions of patients with TN. L (R), left (right) hemisphere.
