# Supplementary material for: Cerebral perfusion alterations in patients with trigeminal neuralgia as measured by pseudo-continuous arterial spin labeling
Source: Front Neurosci. 2022 Dec 16;16:1065411. doi: 10.3389/fnins.2022.1065411 (PMC9807247; doi:10.3389/fnins.2022.1065411)
Supplement: Supplementary file 1 [file Table_1.DOC]

**Supplementary Table 1.** Brain regions with cerebral blood flow differences in patients with trigeminal neuralgia compared to matched healthy controls using CBF normalized by z-transformation

| **Brain regions** | **Cluster size**  **(voxels)** | **Peak MNI coordinate** | | | **Z score** |
| --- | --- | --- | --- | --- | --- |
|  |  | **X** | **Y** | **Z** |  |
| *Increased rCBF regions* |  |  |  |  |  |
| Thalamus* | 424 | -12 | -18 | 8 | 6.0 |
| Insula, L* | 445 | -38 | 10 | -8 | 4.9 |
| Middle frontal gyrus, L* | 454 | -34 | 60 | -16 | 4.5 |
| Middle frontal gyrus, R* | 844 | 30 | 66 | -14 | 5.3 |
| Fusiform, R  Vermis | 60  78 | 36  2 | -22  -62 | -36  -2 | 4.4  4.2 |
| *Decreased rCBF regions* |  |  |  |  |  |
| Middle temporal gyrus, R  Calcarine, L  Superior temporal gyrus, R  Postcentral, R  Precentral, R | 32  33  33  96  40 | 58  0  64  44  36 | -54  -94  -26  -32  -14 | 0  2  12  56  58 | -4.0  -3.8  -4.0  -4.5  -4.9 |

All results were taken at cluster level *P* < 0.05 with an uncorrected cluster forming threshold of *P* < 0.001. *Regions survived gaussian random field correction with a voxel value of *P* < 0.001 and a corrected cluster significance of *P* < 0.05. MNI, Montreal Neurological Institute; L, left hemisphere; R, right hemisphere.
