# Supplementary figures and images for: Cerebral perfusion alterations in patients with trigeminal neuralgia as measured by pseudo-continuous arterial spin labeling
Source: Front Neurosci. 2022 Dec 16;16:1065411. doi: 10.3389/fnins.2022.1065411 (PMC9807247; doi:10.3389/fnins.2022.1065411)

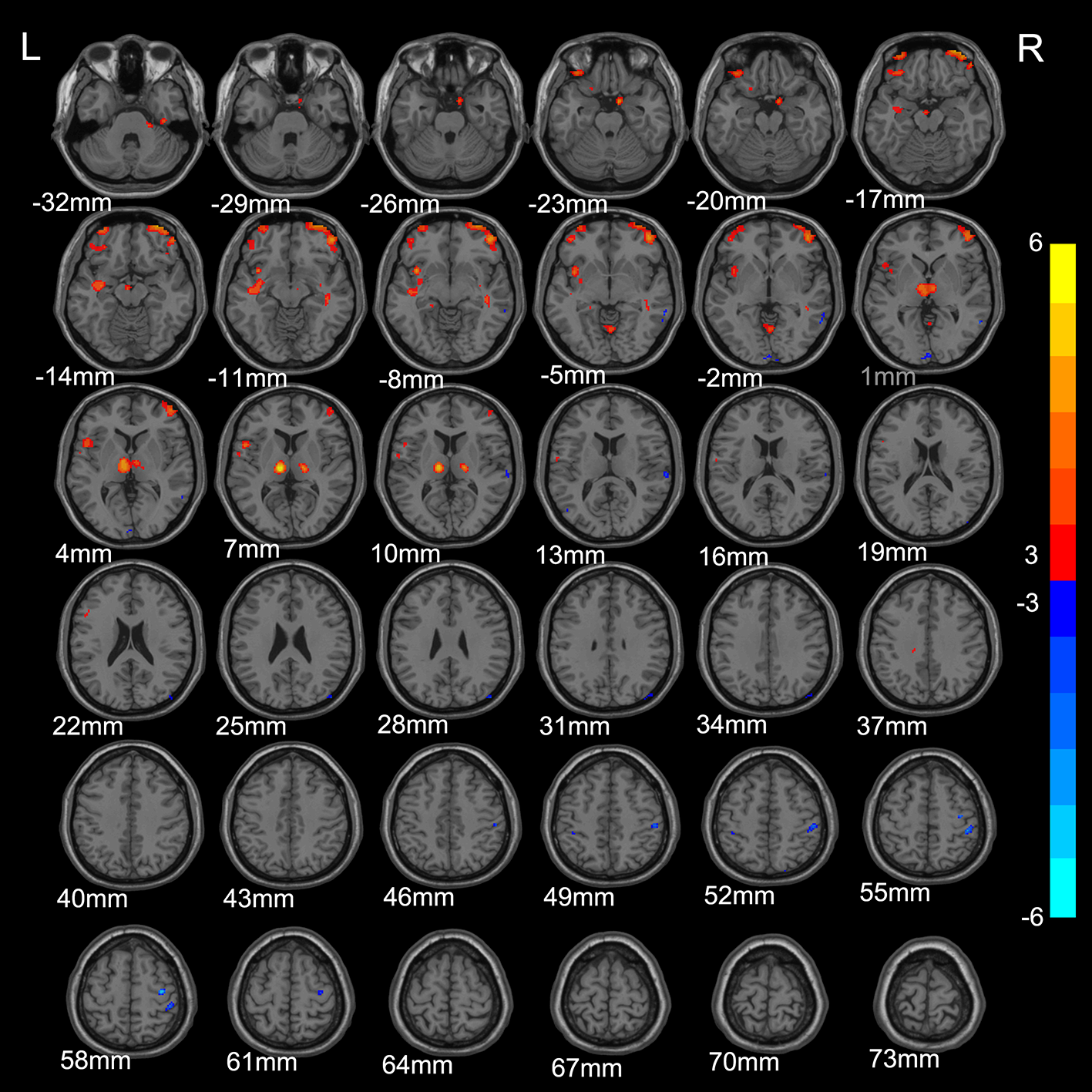

Supplement: Supplementary Figure 1 — Results of altered regional cerebral blood flow (rCBF) in patients with trigeminal neuralgia (TN) compared with healthy controls (HCs). Results were taken at cluster level P < 0.05, with a cluster forming threshold of P < 0.001 uncorrected. The cold and warm colors indicated that the cerebral blood flow (CBF) value decreased and increased, respectively, in the brain regions of patients with TN. L (R), left (right) hemisphere. [file Image_1.TIF]
